# Synthesis of a Novel Fluorescent Sensor Bearing Dansyl Fluorophores for the Highly Selective Detection of Mercury (II) Ions

**DOI:** 10.3390/molecules15031798

**Published:** 2010-03-12

**Authors:** Nantanit Wanichacheva, Supranee Watpathomsub, Vannajan Sanghiran Lee, Kate Grudpan

**Affiliations:** 1 Department of Chemistry, Faculty of Science, Silpakorn University, Nakorn Pathom 73000, Thailand; E-Mails: watpathomsub.s.j@gmail.com (S.W.); 2 Department of Chemistry and Center for Innovation in Chemistry, Faculty of Science, Chiang Mai University, Chiang Mai 50200, Thailand; E-Mails: vannajan@gmail.com (V.S.L.); kgrudpan@gmail.com (K.G.)

**Keywords:** mercury sensor, fluoroionophore, Hg^2+^-selectivity, fluorescent sensor

## Abstract

A new macromolecule possessing two dansyl moieties and based on 2-[4-(2-aminoethylthio)butylthio]ethanamine was prepared as a fluorescent sensor and its mercury sensing properties toward various transition metal, alkali, and alkali earth ions were investigated. The designed compound exhibited pronounced Hg^2+^-selective ON-OFF type fluorescence switching upon binding. The new compound provided highly selective sensing to Hg^2+^ in acetonitrile-water solvent mixtures with a detection limit of 2.49 x 10^-7 M or 50 ppb. ^The molecular modeling results indicated that ions-recognition of the sensor originated from a self assembly process of the reagent and Hg^2+^ to form a helical wrapping structure with the favorable electrostatic interactions of Hg^2+^coordinated with sulfur, oxygen, nitrogen atoms and aromatic moieties.

## 1. Introduction

Mercury is a highly toxic and hazardous environmental contaminant, even at low levels [1]. To allow mercury detection with rapid, convenient and inexpensive methods, a fluorescent sensor can be useful. A number of macromolecules have been proposed and prepared as new fluorescent sensors due to their high selectivity and sensitivity for the detection of metal cations, including mercury [2,3,4,5,6,7,8]. Recently many fluorescent mercury ionophores have been designed for Hg^2+^-sensing such as calixarene [9], hydroxyquinolines [10,11], azines [12], cyclams [13,14,15], diazacrown ethers [16] dioxocyclams [17], diazatetrathia crown ethers [18], and most of these studies have shown that nitrogen, oxygen and sulfur atoms present in the ionophores can promote the coordination of Hg^2+^ [10,11,12,13,14,15,16,17,18]. However, some of these sensors have drawbacks in term of synthetic difficulty, high cost of starting materials or lack of selectivity. 

**Figure 1:**
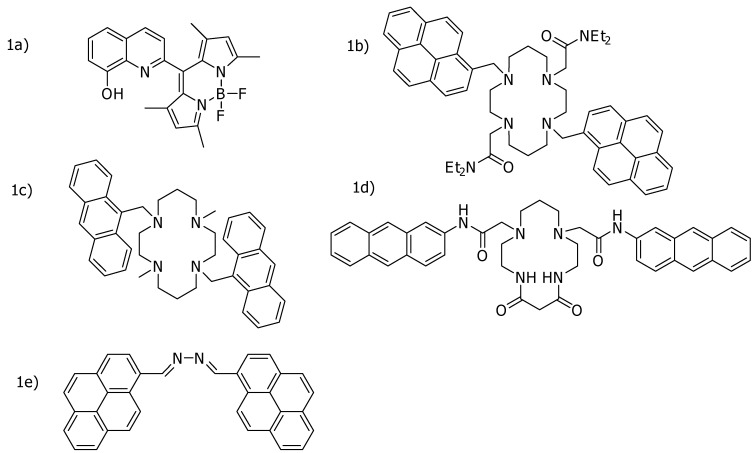
Chemical structures of some mercury sensors.

Although many fluorescent mercury sensors have been designed for Hg^2+^-sensing, they often suffered from some interference by foreign ions, particularly silver (Ag^+^), copper (Cu^2+^) and cadmium (Cd^2+^) due to their similar chemical behaviors to Hg^2+^ [10,12,13,14,17,18,19,20,14,17]. For example, Moon and co-workers [10] prepared a fluorescence sensor based upon 8-hydroxyquinoline (Figure 1a) as a Hg^2+^-fluorescence sensor with the detection limit of 5 × 10^-6^ M. However, this sensor system also displayed moderate sensitivity to Cu^2+^ ions. Park and co-workers [14] as well as Youn and co-workers [20] prepared Hg^2+^-fluorescence sensors based on cyclam moieties. However, a cyclam derivative having diametrically disubstituted pyrene fluorophores (Figure 1b) [14] exhibited chemosensing behavior toward both Hg^2+^ and Cu^2+^, with a detection limit of 1.45 × 10^-6^ M for Hg^2+^. In addition, a diametrically disubstituted bis(anthrylmethyl) derivative of 1,8-dimethylcyclam (Figure 1c) presented by Youn and co-workers [20] exhibited pronounced Hg^2+^ and Cd^2+^ selective fluorogenic behaviors and the sensor provided a detection limit of 3.8 × 10^-6^ M for Hg^2+^. Song and co-workers [17] prepared a fluorescence sensor based upon the dioxocyclam moiety. They observed that the dioxocyclam derivative bearing anthrylacetamide moieties (Figure 1d) exhibited both Hg^2+^ and Cu^2+^ sensing with a detection limit of 7.8 × 10^-6^ M for Hg^2+^. Martinez and co-workers [12] introduced a fluorescence sensor based on the azine moiety. They found that 1,4-bis(1-pyrenyl)-2,3-diaza-1,3-butadiene (Figure 1e) could be employed as a fluorescence probe for both Hg^2+^ and Cu^2+^ with a detection limit of 3.4 × 10^-6^ M for Hg^2+^. For the selective recognitions presented above, ligands with only nitrogen binding sites might not be sufficient for the discrimination of potential interfering ions such as Cu^2+^ and Cd^2+^. Consequently, they are not effective as a selective Hg^2+^-fluorescence sensor. Figure 1 shows the chemical structures of some of the mentioned mercury sensors. 

In this study, the design concept for the sensor is based on the fundamental requirements for the selective host-guest interactions in supramolecular chemistry. The major motivation of our work is the design and synthesis of mercury sensors with high sensitivity and selectivity but with a significantly reduced synthetic effort. We have focused on utilizing the new ligand, 2-[4-(2-aminoethylthio) butylthio]ethanamine (**3**), as an acyclic host which offers a highly flexible ligand system with appropriately located donor atoms that can self assemble around the guest molecule due to the favorable electrostatic interactions. We have chosen dansyl as a fluorophore for the construction of the chemosensor due to its strong fluorescence, relatively long emission wavelength in the visible region and structural flexibility for derivatization [9,21,22,23].

Herein, we wish to report that the strong fluorescence of the dansyl moieties attached to 2-[4-(2-aminoethylthio)butylthio]ethanamine in the novel compound **4** is quenched considerably and in a selective manner upon complexation with Hg^2+^. The designed compound exhibits a high Hg^2+^-selectivity in comparison with foreign ions (Ag^+^, Ba^2+^, Ca^2+^, Cd^2+^, Co^2+^, Cu^2+^, Fe^2+^, Fe^3+^, Mn^2+^, Na^+^,Ni^2+^, Pb^2+^and Zn^2+^) in acetonitrile:water solutions. Compound **4** offers sufficiently low detection limits for the determination of sub-micromolar concentrations hazardous Hg^2+^ ions found in environmental and biological samples such as edible fish [24].

## 2. Results and Discussion

**Scheme 1:**
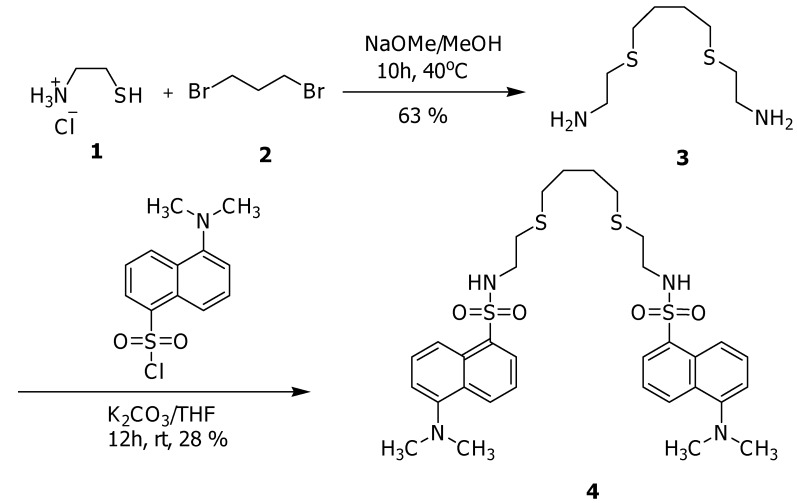
Synthesis of **4**.

Fluoroionophore **4** was prepared using a conventional two-step synthesis as outlined in Scheme 1. 2-[4-(2-aminoethylthio)butylthio]ethanamine (**3**) was prepared by alkylation of cysteamine hydrochloride (**1**) with 1,4-dibromobutane (**2**). Then compound **4 **was obtained by reaction of **3 **with 5-(dimethylamino)naphthalene-1-sulfonyl chloride. Compound **4** is a podant, acyclic host with pendant binding sites [25], containing two sulfur atoms and two nitrogen atoms which are covalently bound to two dansyl subunits. We expect that the selective binding will take place through electrostatic interactions between the sulfur and nitrogen atoms of the ligand and Hg^2+^, as these interactions are well known [10,11,12,13,14,15,16,17,18].

### 2.1. Molecular modeling studies

**Figure 2:**
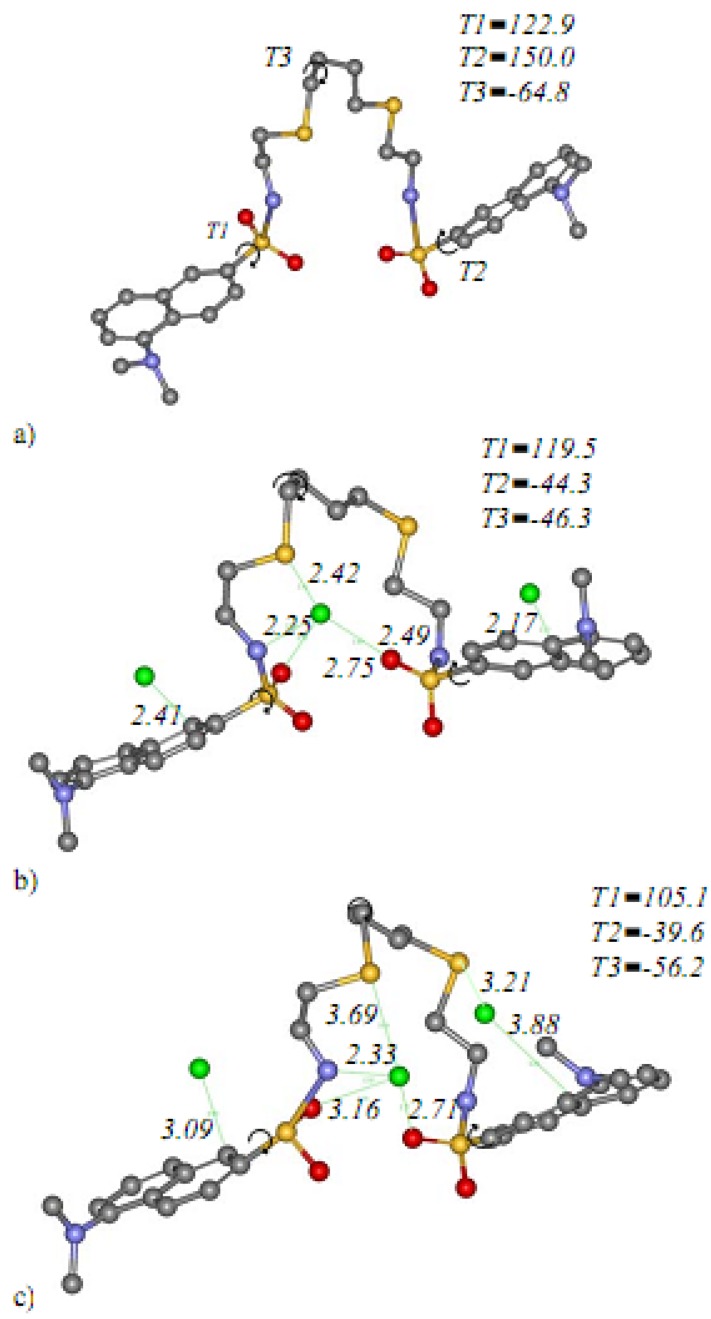
a) The lowest optimized structure from conformational search b) the host (compound **4**) – guest (3Hg^2+^) structure from MDs in acetonitrile:water (95:5) using implicit distance-dependent dielectrics of 38.43 c) the lowest optimized host - guest structure from MDs and optimized with local density approximation (LDA) of local functional PWC with implicit distance-dependent dielectrics of 38.43. The distances in Å between host and guest were labeled.

An attempt to theoretically estimate the binding mode diversity on the molecular recognition seen in the prominent complexation of **4** and Hg^2+^ is described. The helical structure of compound **4** was initially obtained from semi-empirical method using AM1 in the gas phase. Then this structure was subjected to conformation analysis by varying the possible 17 torsions and the lowest energy conformation was obtained in Figure 2a with the torsions *T1: C1-C2-S14-N44,* *T2: C17-C18-S30- N33*, *T3: S41-C40-C39-C38* of 122.9, 150.0, and -64.8, respectively. The absorptions of three Hg^2+^ loading structures were further investigated on the lowest conformation as illustrated in Figure 2b. The dynamics simulation was performed to obtain the possible complexes between the host and guest molecules in acetonitrile-water (95:5) using implicit distance-dependent dielectrics of 38.43 with CHARMm force field. The lowest complexation energy conformation was selected for optimization using density functional theory of local functional PWC [26] with implicit distance-dependent dielectrics of 38.43. The final structure obtained for compound **4** - 3Hg^2+^ complex is shown in Figure 2c. Each of the Hg^2+^ ions was located at the helical loops and coordinated with S, O, N, and aromatic moieties with the favorable electrostatic interactions. The binding energy can be estimated from the difference between the energy of the complex and the sum of individual **4** and 3Hg^2+^ energies. The low complexation energy of -128.621 kcal/mol showed the stability of this complex.

The molecular modeling results (Figure 2c) indicated that ions-recognition of the sensor originated from a self assembly process of compound **4** and 3Hg^2+^ from the favorable electrostatic interactions of Hg^2+^ coordinated with S, O and N atoms as well as aromatic moieties (ion-dipole interaction with the middle Hg^2+^ and cation-π interaction with the other two Hg^2+^ ions) to form a helical wrapping structure.

### 2.2. Sensitivity studies

In this study, the effects of water on the fluorescence emission of **4** in the absence and presence of Hg^2+^ were systematically investigated in acetonitrile solutions in order to optimize the conditions for practical applications in environmental and biological samples. 

**Figure 3:**
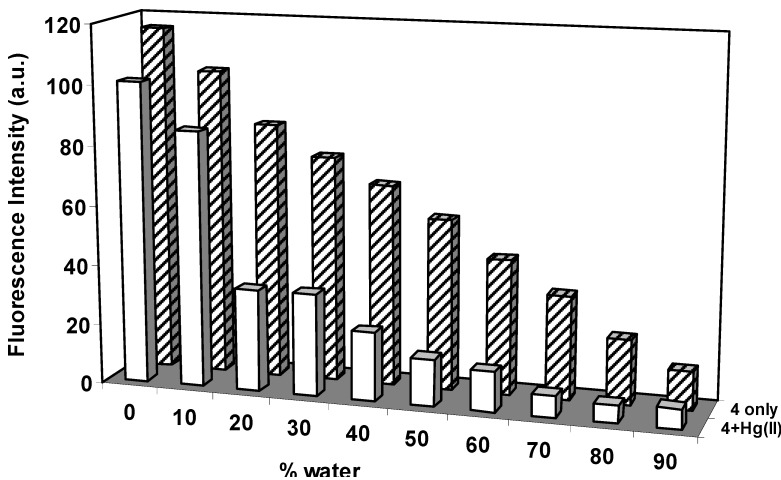
Fluorescence intensity changes of **4** (0.5 μM) as a function of water content in aqueous acetonitrile solution at 515 nm in the absence and presence of Hg^2+^ (20 equiv), λ_ex _338 nm.

The effect of water content on the fluorescence behavior of **4** in acetonitrile solutions is shown in Figure 3. The fluorescence emission of **4** was found to be strongly dependent on the presence of water in the aqueous acetonitrile solutions. This result illustrated that when the concentration of water increased, the fluorescence emission intensity of **4** decreased progressively. In the low water concentration range, a similar decrease in the response of **4** in the presence of 20 equivalents of Hg^2+^ was observed, but with much larger changes compared to the high water concentration region. Based on this observation, we focused on the fluorescence behaviors of **4** in response to the various metal ions in 80:20 acetonitrile:water solution. 

**Figure 4:**
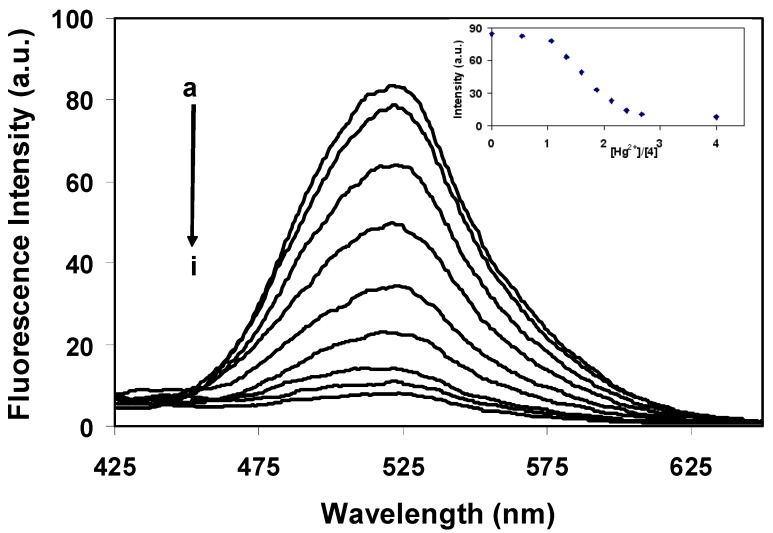
Fluorescence emission spectra (λ_ex_ 338 nm) of **4** (0.25 μM) in 80:20 CH_3_CN:H_2_O as function of [Hg^2+^]. a: 0 M, b: 0.27 μM, c: 0.33 μM, d: 0.40 μM, e: 0.47 μM, f: 0.53 μM, g: 0.60 μM, h: 0.67 μM, i: 1.00 μM.

To elucidate the quantitative binding affinity of **4**, fluorescence titrations of **4** with Hg^2+^ were carried out. Figure 4 shows the fluorescence spectra obtained from **4** in the absence and presence of Hg^2+^ in 80:20 acetonitrile-water.

When an ion-complexation was operative, an ON-OFF switching occurred as indicated by the fluorescence emission behavior. In the absence of Hg^2+^ ions, the fluorescence response was at a maximum and the response decreased as the mercury concentration was increased. When the added mercury acetate attained a concentration approximately more than three times higher than that of **4**, more than 90 % quenching of initial fluorescence of **4** was observed and the fluorescence response reached a minimum point. The inset of Figure 4 shows the break around 3-4 equiv of Hg^2+^ which could suggest a 1:3 or 1:4 stoichiometry for the **4**-Hg^2+^ complex system [18]. Further studies by molecular modeling using Material Studio 4.3 suggested that the complex formation having the 1:3 (host **4**: guest Hg^2+^) stoichiometry was appropriate and provided the most stable complexation (-128.621 kcal/mol) while the complex of 1:4 (host **4**: guest Hg^2+^) could not be formed. The association constant, *K*_assoc_, was determined by nonlinear curve fitting of the signal changes in the fluorescence titration results [10,14,27,28]. It was found to be 1.15 × 10^19^ M^-3^ and the 1:3 complex formation of **4**-Hg^2+^ was suggested. 

A similar quenching behavior upon Hg^2+^ binding was previously observed in many mercury fluorescence sensors [10,16,17,18,20], and a mechanism such as the photo-induced electron transfer (PET) process was suggested [9,20]. For this system, the fluorescence quantum yield (φ_f_) of **4** in acetonitrile was found to be 0.55, using quinine sulfate as a reference [29]. However, the φ_f_ of the 1:3 complex formation of **4**-Hg^2+^was found to be 0.54. An insignificant change of the quantum yield indicated that the quenching mechanism upon Hg^2+^ binding to **4** did not occur via the PET process for this system. The effective fluorescence quenching of **4** might be due to the inherent quenching nature of Hg^2+^ ions as suggested by Chang and co-workers [15,17,18]. 

The data collected in Figure 4 provided a good linear correlation between the emission response and Hg^2+^ concentration over a range of 53–120 ppb, which is sufficient for the detection of sub-micromolar concentrations of Hg^2+^ found in many biological systems such as edible fish [24]. The detection limit of **4** as a fluorescent sensor for the analysis of Hg^2+^ was determined from the plot of the fluorescence intensity as a function of the Hg^2+^ concentrations [30] and was found to be approximately 50 ppb. 

### 2.3. Selectivity studies

**Figure 5:**
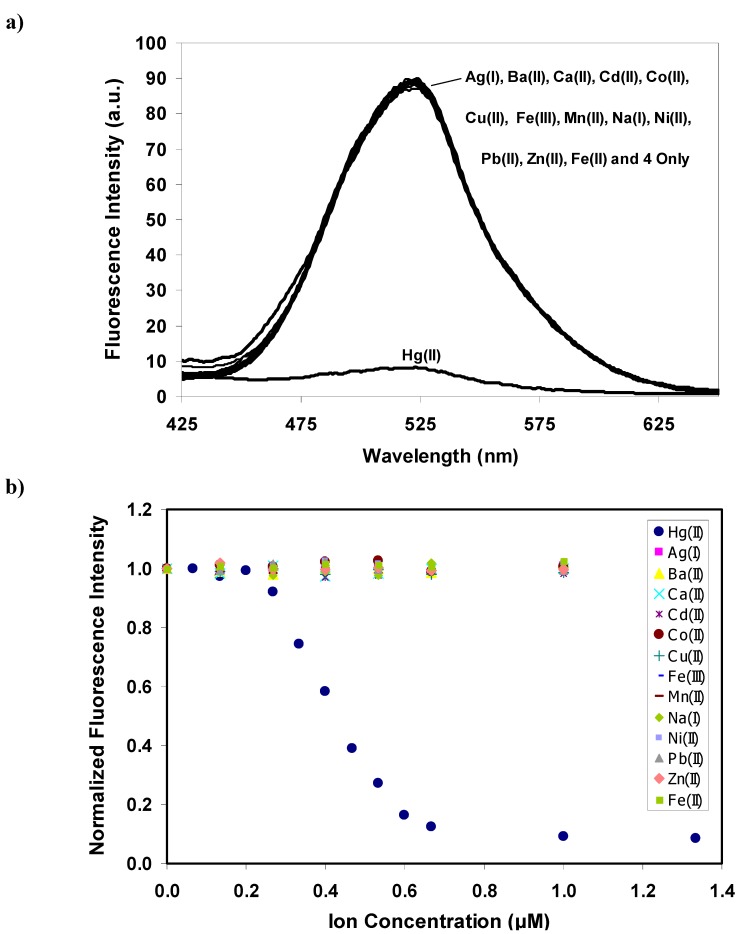
a) Fluorescence spectra (*λ*_ex_ = 338 nm) of **4** (0.25 μM) with addition of acetate salts of Hg^2+^, Ag^+^, Ba^2+^, Ca^2+^, Cd^2+^, Co^2+^, Cu^2+^, Fe^2+^, Fe^3+^, Mn^2+^, Na^+^,Ni^2+^, Pb^2+^ and Zn^2+ ^(1.00 μM) b) Normalized emission intensity (515 nm) of **4** (0.25 μM) versus the concentration of various metal ions in 80:20 CH_3_CN:H_2_O.

Selectivity studies of **4** were performed in 80:20 acetonitrile-water solutions by recording the fluorescence spectra of the solutions of **4** after the addition of each representative metal ions. Figure 5 shows the dependence of the fluorescence intensity of **4** as a function of cation concentrations for Hg^2+^, transition-metal, heavy metal, alkali earth and alkali ions including Ag^+^, Ba^2+^, Ca^2+^, Cd^2+^, Co^2+^, Cu^2+^, Fe^2+^, Fe^3+^, Mn^2+^, Na^+^, Ni^2+^, Pb^2+^ and Zn^2+^. Herein, the sensitivity studies of **4** were performed in 80:20 acetonitrile:water solution by a similar method to the Separate Solution Method (SSM) used in ion-selective electrode applications [31]. This method involves the measurement of a series of separate solutions, each containing only a salt of the determined ion [31].

The selectivity studies clearly demonstrated the high selectivity of **4** to Hg^2+^ in comparison with the other cations. The results showed that the fluorescence intensity of **4** decreased as a function of added Hg^2+^ until the minimum point was reached, beyond which it was constant up to the maximum concentration tested. In contrast, the fluorescence response of **4** promoted a small change after the addition of other foreign ions under identical conditions. It should be noted here that **4** showed high selectivity for Hg^2+^ over Ag^+^, Cu^2+^ and Cd^2+^ which are potential competitors [12,13,14,17,18,19,20,14,17].

We have therefore demonstrated that our new ligand, 2-[4-(2-aminoethylthio)butylthio]ethanamine, meets the fundamental requirements for the selective host-guest interactions in supramolecular chemistry for chelating Hg^2+^. The sensor fabricated from the ligand with appropriately located donor atoms (S, N, O) can provide selective self assembly around Hg^2+^ due to the favorable electrostatic interactions (ion-dipole interactions). Due to the advantages of high selectivity and synthetic simplicity, 2-[4-(2-aminoethylthio)butylthio]ethanamine could therefore be utilized as a potential mercury ligand for future applications.

## 3. Experimental

### 3.1. General

Barium acetate was purchased from Sigma-Aldrich. Cadmium acetate was purchased from Merck and cobalt acetate was purchased from Prolabo. All other reagents and solvents for synthesis were purchased from Fluka Chemical Corporation and were used as received, unless stated otherwise. Aqueous solutions were freshly prepared using high-purity Millipore deionized water (18 MΩ.cm).

NMR spectra were obtained with a Bruker Avance spectrometer operating at 300 MHz for proton and 75 MHz for ^13^C. All NMR spectra were obtained on CDCl_3_ solutions. Mass spectra were performed by a ThermoElectron LCQ-DECA-XP, electrospray ionization ion trap mass spectrometer. 

Fluorescence measurements: Fluorescence emission and excitation spectra of **4** were obtained with a Perkin Elmer Luminescence spectrometer LB50 with the use of 80:20 acetonitrile-water as solvent, with an excitation wavelength 338 nm. Typical solutions were made with fluoroionophore **4** (0.25 μM). Metal ions were added as the acetate salts. Fluorescence was measured as a function of metal ions concentration. For selectivity studies, fluorescence intensity was determined at a fixed wavelength (515 nm).

### 3.2. Synthesis of 2-[4-(2-aminoethylthio)butylthio]ethanamine (***3***)

In a round bottom flask, sodium methoxide (0.68 g, 12.00 mmol) was dissolved in dry methanol (3 mL). Cysteamine hydrochloride (1.14 g, 10.03 mmol) was added to the solution. The solution was then stirred for 30 min where upon 1,4-dibromobutane (0.5 mL, 4.14 mmol) was added. This solution was then stirred for an additional 10 h at 40 °C under an argon atmosphere. The solvent was subsequently removed by a rotary evaporator. Aqueous sodium hydroxide solution (30 % w/v, 15 mL) was added to the residue and the resulting solution was slowly stirred overnight. Then, the solution was extracted three times each with 20 mL of dichloromethane. The dichloromethane phase was collected and washed once with 60 mL of distilled water and then dried over anhydrous Na_2_SO_4_. The dichloromethane was removed under vacuum to give 0.54 g of the product as yellow oil (63% yield). The product was used without further purification. ^1^H-NMR (CDCl_3_) δ 1.58 (s, 4H), 1.63-1.75 (m, 4H), 2.43-2.57 (m, 4H), 2.57-2.71 (m, 4H), 2.81-2.91 (m, 4H); ^13^C-NMR (CDCl_3_) δ 32.5 (2CH_2_), 35.2 (2CH_2_), 39.2 (2CH_2_), 44.4 (2CH_2_); HRMS calcd for C_8_H_21_N_2_S_2_ (M+H)^+^ 209.1146 m/z, found 209.1073 m/z.

### 3.3. Synthesis of compound 4

In a round bottom flask, 2-[4-(2-aminoethylthio)butylthio]ethanamine (**3**) 0.10 g, 0.48 mmol) and K_2_CO_3_ (0.26 g, 1.92 mmol) were stirred in dry tetrahydrofuran (7 mL) for 30 min under an argon atmosphere. 5-(Dimethylamino)naphthalene-1-sulfonyl chloride (0.32g, 1.20 mmol) was added and the reaction mixture was stirred for 12 h at room temperature. The potassium carbonate was filtered off and carefully washed with dichloromethane. The solvent was removed under vacuum. The crude product was purified by preparative thin layer chromatography (CH_2_Cl_2_:MeOH 99:1 eluent) to afford 92.2 mg of a yellow oil in 28% yield. *R*_f_ = 0.29 (CH_2_Cl_2_:MeOH 99:1); ^1^H-NMR (CDCl_3_) δ 1.31-1.41 (m, 4H), 1.61 (s, 4H), 2.06-2.24 (m, 4H), 2.49 (t, *J* = 6.3 Hz, 4H), 2.89 (s, 12H), 3.02 (q, *J* = 6.3 Hz, 4H), 7.18 (d, *J *= 7.5 Hz, 2H), 7.47-7.62 (m, 4H), 8.20-8.32 (m, 4H), 8.54 (d, *J* = 8.4 Hz, 2H); ^13^C-NMR (CDCl_3_) δ 28.0 (2CH_2_), 30.6 (2CH_2_), 31.7 (2CH_2_), 41.7 (2CH_2_), 45.4 (4CH_3_), 115.3 (2C), 118.6 (2C), 123.2 (2C), 128.5 (2C), 129.7 (2C), 130.6 (2C), 129.6 (2C), 129.8 (2C), 134.5 (2C), 152.0 (2C); HRMS calcd for C_32_H_43_N_4_O_4_S_4_ (M+H)^+^ 675.2167 m/z, found 675.2000 m/z.

### 3.4. Computational modeling of complex structure with mercury

Two end chains [-SO_2_C_10_H_6_(CH_3_)_2_] of compound **4** were obtained from the X-ray crystal structure (PDB ID: 1BDA) where the straight chain in the middle was built using Material Studio 4.3. Then, this initial structure was optimized using AM1 method with RHF in the gas phase. The optimized structure was submitted to the conformers module within the same program software package to generate conformers of the molecule using Boltzman jump method with the number of perturbation per jump = 50 at 5000 K [32,33,34]. All possible 17 torsion angles of compound **4** were rotated with 10 Å torsion angle step. The lowest energy conformer was initially obtained as Figure 1a). Host-guest calculation was performed with three Hg^2+^ ions loading (according to experimental results) where each Hg^2+ ^ion was positioned about the middle of the three helical loops. These systems were minimized and dynamics simulation in acetonitrile:water (95:5) was performed using implicit distance-dependent dielectrics of 38.43 with CHARMm force field. The formal charge for Hg^2+ ^was assigned. Molecular dynamics (MDs) was obtained at the constant temperature at 300 K 100 ps with constraint force of 0.01 kcal/mol/Å^2^ under NVT ensemble. Time step of 1 fs was used in all simulations. The MDs structure was analyzed in detail in Discovery Studio Version 1.7 program package. The lowest complexation energy of the host-guest structure from the dynamics simulation was further optimized with more accurate calculation method using density functional theory with local density approximation (LDA) of local functional PWC [26] with implicit distance-dependent dielectrics of 38.43 and the final structure of complexation of **4** and 3Hg^2+^ ions complex was obtained.

## 4. Conclusions

In summary, we have discovered a new mercury fluoroionophore that exhibited high selectivity for Hg^2+^ over a wide range of foreign ions, but with a significantly reduced synthetic effort. Compound **4** consists of two dansyl fluorophores covalently bound to 2-[4-(2-aminoethylthio)butylthio]ethanamine. The sensor was prepared by a conventional two-step synthesis. The Hg^2+^-selective fluorescence quenching of **4** was observed in aqueous acetonitrile solutions with the detection limit of 2.49 × 10^-7^ M or 50 ppb. The molecular design presented here could serve as an alternative mercury fluorometric sensor due to the advantage of sensitivity, selectivity and synthetic simplicity. We have therefore developed a highly selective and sensitive fluoroionophore for Hg^2+^ detection and the ready synthetic access to the dansyl-based fluoroionophore could make it an attractive mercury sensor for many potential applications.
